# Ammidin ameliorates myocardial hypoxia/reoxygenation injury by inhibiting the ACSL4/AMPK/mTOR-mediated ferroptosis pathway

**DOI:** 10.1186/s12906-023-04289-x

**Published:** 2023-12-15

**Authors:** Yue Han, Hui Yuan, Fengxiang Li, Yueying Yuan, Xuezhi Zheng, Xudong Zhang, Jian Sun

**Affiliations:** 1 Collaborative Innovation center of development and application of North medicine resources in Mudanjiang City, Muandanjiang, China; 2 School of Basic Medicine of Mudanjiang Medical University, Department of Physiology, Mudanjiang Medical University, No.3 tong xiang street, Ai min district, Muandanjiang, China

**Keywords:** Ammidin, ACSL4/AMPK/mTOR signaling pathway, Glutathione peroxidase 4, Network pharmacology, Ferroptosis

## Abstract

**Objective:**

The aim of the present study was to investigate the therapeutic effect of ammidin on hypoxia/reoxygenation (H/R) injury in primary neonatal rat cardiomyocytes by observing the role of ferroptosis in the process of H/R injury, and to verify its target and regulatory signaling pathways.

**Methods:**

The network pharmacology analysis was used to predict the biological processes, core targets and related signaling pathways of *Angelica dahurica* in the treatment of ferroptosis. Cell viability was assessed using live cell imaging and cell counting kit-8. Lactate dehydrogenase (LDH), reactive oxygen species (ROS) production, and malondialdehyde (MDA), superoxide dismutase (SOD) and mitochondrial membrane potential (MMP) content were determined to assess the level of ferroptosis. Western blotting was performed to measure protein expression.

**Results:**

Network pharmacology predicted that Acyl-CoA synthetase long chain family member 4 (ACSL4) was highly associated with myocardial H/R injury in the intersection of *Angelica dahurica* and ferroptosis. The top three active components of *Angelica dahurica* were found to be mandenol, alloisoimperatorin and ammidin, among which ammidin was found to have the strongest binding to the target proteins of the ACSL4/AMPK/mTOR pathway. H/R reduced the viability of cardiomyocytes, while the inhibition of ferroptosis by ferrostatin-1 alleviated the H/R-induced inhibition of cardiomyocyte viability. This was evidenced by the increased cell viability, SOD release, MMP level and glutathione peroxidase 4 (GPX4) protein expression, as well as the decreased LDH and MDA release and ROS production and ACSL4 protein expression (*P* < 0.05). To verify the existence of ferroptosis in myocardial hypoxia/reoxygenation injury. In addition, ammidin increased cell viability and GPX4 protein expression (*P* < 0.05), decreased ROS generation, and MDA and MTT expression (P < 0.05), then inhibited ferroptosis, and finally alleviated myocardial H/R injury by regulating the ACSL4/AMPK signaling pathway.

**Conclusions:**

Network pharmacology was used to predict the correlation between ammidin and ferroptosis following myocardial H/R injury. It was demonstrated that ammidin may regulate ferroptosis by inhibiting the ACSL4/AMPK/mTOR signaling pathway and reduce H/R injury in cardiomyocytes.

## Introduction

**Fig. 1:**
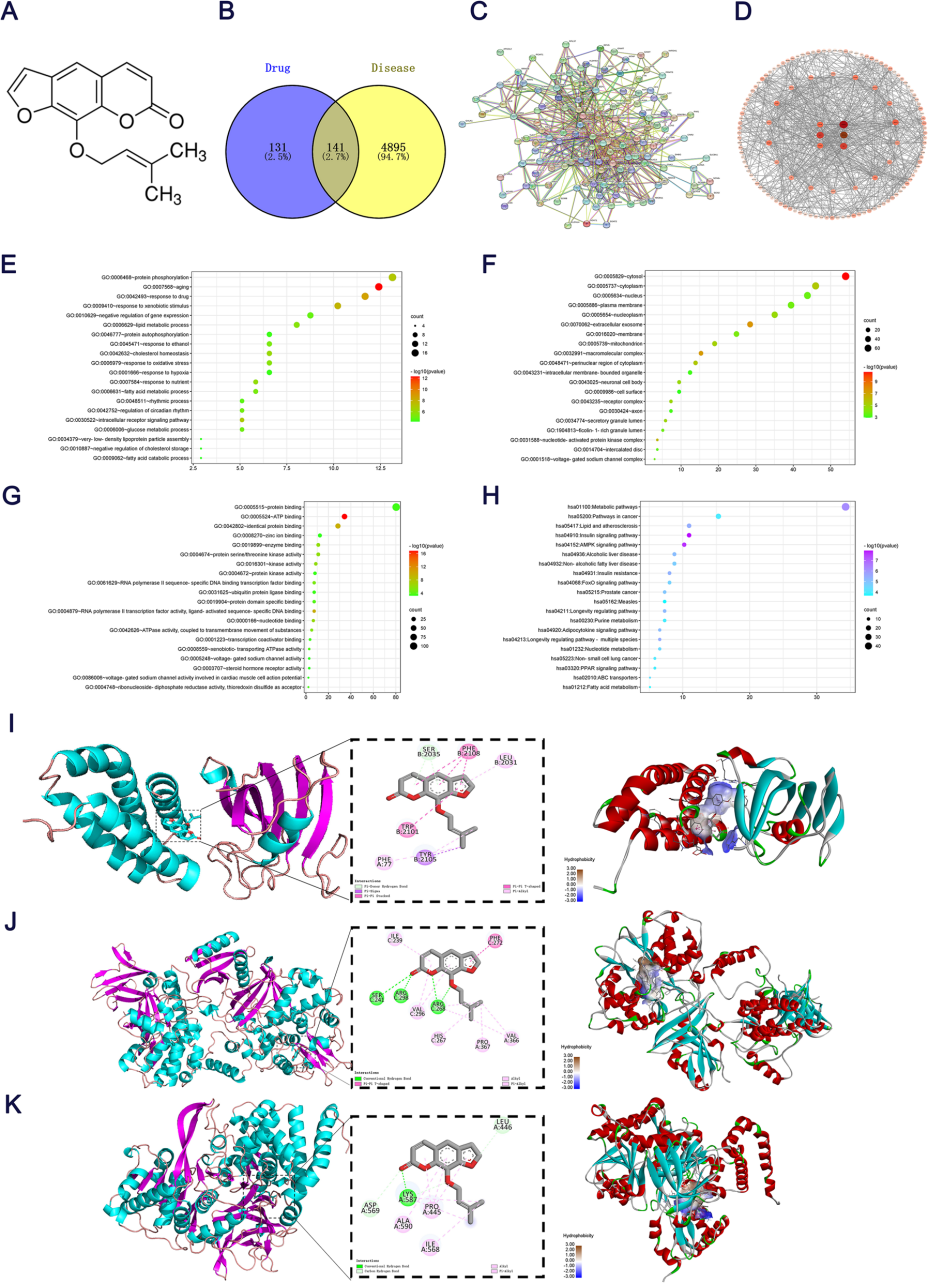
Network pharmacology. **A** Structural formula for ammidin. **B** Venn diagram of *Angelica dahurica* and ferroptosis. **C** Cross-PPI networks. **D** PPI network core screening. GO results for **E** biological processes, **F** cellular components and **G** molecular function. **H** Results of KEGG enrichment analysis (*P* < 0.05). **I** The left picture shows the overall picture of the interaction between protein mTOR and small molecule ammidin, and the right picture is a 3D view of mTOR and ammidin. **J** AMPK and ammidin. **K** ACSL4 and ammidin. PPI, protein-protein interactions; KEGG, Kyoto Encyclopedia of Genes and Genomes; ACSL4, Acyl-CoA synthetase long chain family member 4

Myocardial ischemia-reperfusion injury (MIRI) means that after acute coronary artery occlusion, reperfusion leads to more serious myocardial injury than ischemia itself [1], such as myocardial dysfunction, structural damage and mitochondrial dysfunction [2]. At present, there are drugs for the treatment of MIRI,such as nitroglycerin and curcumin, but they are not very effective [3].. Modern drug development is deeply influenced by natural products, particularly cardioprotective and anti-ischemic drugs [4]. As the main component of *Angelica dahurica*, ammidin (Fig. 1A) exerts a wide range of cardiovascular protection effects [5]. However, the mechanism of the application of ammidin in myocardial hypoxia/reoxygenation (H/R) injury remains unknown [6]. Several studies have reported that ammidin was predicted to prevent the occurrence of cardiovascular diseases by inhibiting hypertension, myocardial fibrosis based on network pharmacology [7]. However, the correlation between ammidin and ferroptosis has not yet been predicted by network pharmacology.

Unlike other classical non-apoptotic cell death processes, ferroptosis is a type of regulatory cell death, is characterized by mitochondrial contraction and enhanced mitochondrial membrane density (morphology), lipid peroxidation (biochemistry), and a unique set of genes (heredity), [8]. Fer-1 and desferrioxamine have been shown to contribute to the improvement of acute and chronic MIRI-induced heart failure, which is consistent with the idea of targeting ferroptosis as a potential strategy for the prevention of cardiomyopathy [9]. Previous studies have shown that glutathione peroxidase 4 (GPX4) is a peroxidase reductase containing selenium, which can decompose lipid peroxides into non-toxic hydroxyl lipids, and is the core protease that antagonizes ferroptosis [10]. Acyl-CoA synthetase long-chain family member 4 (ACSL4), a member of the ACSL family, is a key regulator of ferroptosis. The overexpression of ACSL4 induces the activation of the ferroptosis pathway [11]. A previous study showed that sevoflurane induced ferroptosis in neurons by regulating the AMPK/mTOR pathway, and that ACSL4 inhibitors also inhibited ferroptosis, indicating that ACSL4 could alleviate ferroptosis by regulating the AMPK/mTOR pathway [12].

The H/R injury model of primary lactating rat cardiomyocytes was used in the present study. By intervening with protein inhibitors, the target and signaling pathways of ammidin were explored, in the hope that it will provide experimental support and a theoretical basis for the clinical treatment of myocardial ischemic diseases.

## Materials and methods

### Materials

Neonatal SD suckling mice aged 1–3 days (Medical Research Center, Mudanjiang Medical University), sodium dithionite (Na_2_S_2_O_4_; Sigma-Aldrich; Merck KGaA), PRGL493, ammidin, ferrostatin-1 (Fer-1; MedChemExpress), enhanced cell counting kit-8 (CCK-8), lactate dehydrogenase (LDH) and mitochondrial membrane potential (MMP) assay kit with 1,1′,3,3′-tetraethyl-5,5′,6,6′-tetrachloroimida Carbyan.

ine iodide (JC-1) were analyzed using a standard biochemistry panel (Beyotime Institute of Biotechnology). Malondialdehyde (MDA) and superoxide dismutase (SOD) were measured using commercially available kits (Nanjing Jiancheng Bioengineering Institute). Anti-β-actin (cat. no. sc-47,778; dilution, 1:1000) and goat anti-rabbit IgG (cat. no. sc-2004; dilution, 1:20,000) were purchased from Santa Cruz Biotechnology, Inc. Primary antibodies against proteins, including ACSL4 (cat. no. ab155282; dilution, 1:1000), GPX4 (cat. no. ab125066; dilution, 1:1000), AMPK (cat. no. ab32047; dilution, 1:1000), phosphorylated (p-AMPK) (cat. no. ab133448; dilution, 1:1000), mTOR (cat. no. ab134903; dilution, 1:500) and p-mTOR (cat. no. ab109 268; dilution, 1:500), were purchased from Abcam.

### Bioactive ingredients in *Angelica dahurica*

The Traditional Chinese Medicine Systems Pharmacology (TCMSP, https://tcmspw.com/tcmsp.php) database was searched for bioactive ingredients and targets of *Angelica dahurica*, and the target genes of *Angelicae dahuricae* Radix were retrieved from the Encyclopedia of Traditional Chinese Medicine (http://www.tcmip.cn/ETCM/index.php/Home/Index/).

### Targets related to *Angelica dahurica* ingredients and ferroptosis

Ferroptosis targets were found at GeneCards® (genecards.org). The Venny 2.1 platform (https://bioinfogp.cnb.csic.es/tools/venny/) was used to draw the Venn diagram. The protein-protein interactions (PPI) at common targets were completed in the STRING database (https://string-db.org/) with Cytoscape 3.7.0 for visual analysis.

### Enrichment analysis of pathway function

KEGG enrichment analysis, enrichment of gene ontology (GO) biological process, cellular component, and molecular function terms were analyzed using David’s Functional Annotation Chart tool (Version 6.8) [13-15].

### Molecular docking

The three-dimensional (3D) structure of the target protein was downloaded from the RCSB PDB database (https://www.rcsb.org/). PyMOL 2.3.0 was used to analyze the docking results and reveal the 3D protein-ligand complex. AutoDockTools 1.5.6 was used to calculate the affinity between protein and ligand. The hydrogenation and charge of the target protein was calculated, and the conformation with good affinity was observed using PyMOL 2.3.0.

### Culture and grouping of primary cardiomyocytes

Rats were primary cardiomyocytes from 1 to 3-days-old SD rat hearts were extracted and cultured for 52 h, and then randomly divided into 6 groups: i) Normal control group (control), cardiomyocytes were conventionally cultured; ii) H/R group, cardiomyocytes were first cultured for 52 h and 1 h with a final concentration of 4 mM Na_2_S_2_O_4_, and then conventionally cultured; iii) H/R ferroptosis inhibitor (Fer-1) group (H/R + Fer-1), cells were pretreated with 2 μM Fer-1 for 24 h before H/R treatment; iv) H/R + ACSL4 inhibitor (PRGL493) group (H/R + PRGL493), cells were treated with 5 μM PRGL493 during H/R treatment; v) H/R + ammidin group (H/R + A), cells were treated with 20 μM ammidin during H/R treatment; vi) H/R + ACSL4 inhibitor + ammidin group (H/R + PRGL493 + A), cells were treated with 5 μM PRGL493 + 20 μM ammidin during H/R treatment.

### Immunofluorescence staining

Cardiomyocyte slides were incubated with primary (dilution, 1:100) and secondary (dilution, 1:100) antibodies of cardiac troponin T (cTnT) after fixation in 4% pre-cooled paraformaldehyde. Following 4′,6-diamidino-2-phenylindole (DAPI) staining, the images were observed under a laser confocal microscope, and the purity of cardiomyocytes was calculated. The total number of cells and the number of positive cells were counted: Positive cell rate = positive cell number / total number of cells × 100% (*n* = 3).

### Live cell imager

Living cells were imaged using a Leica confocal microscope (Leica Microsystems, Inc.). The beating frequency was observed and the cardiomyocyte area was measured using ImageJ Lab v4.0 (National Institutes of Health). The rate of change of myocardial contraction amplitude = (diastolic area - contractile area) / diastolic area × 100%. ImageJ Lab v4.0 was used for image analysis (*n* = 3).

### Cell proliferation assays

Cardiomyocytes were inoculated in 96-well plates at a density of 5000 cells/well for 48 h, and then treated with different reagents. The optical density value was measured at 450 nm with enzyme label to evaluate cell viability. Dimethyl sulfoxide < 0.1% used for dissolving reagent (*n* = 3).

### Determination of LDH activity

Cardiomyocytes were collected and incubated with LDH release solution for 1 h. The supernatant of cardiomyocytes then reacted with LDH detection solution in the dark for 30 min, and the absorbance was detected at 490 nm (*n* = 3).

### Determination of MDA and SOD activities in the myocardium

MDA content and superoxide dismutase (SOD) activity in myocardial cells was measured by SpectraMax M5 enzyme label and determined according to the manufacturer’s instructions (Beyotime Biotechnology) (*n* = 3).

### Determination of reactive oxygen species (ROS) levels in cardiomyocytes through the 2′,7′-ichlorofluorescein diacetate (DCFH-DA) method

ROS levels in cardiomyocytes were detected by DCFH-DA. The fluorescence images were observed under an Olympus Fluoview FV1,000 microscope (Olympus Corporation). ImageJ was used to quantitatively analyze the fluorescence intensity of images (*n* = 3).

### Determination of mitochondrial membrane potential (MMP)

Following cultivation for 48 h, the cardiomyocyte slides were incubated with 5 μM JC-1 dye at 37 °C for 20 min and observed using a confocal microscope (n = 3).

### Western blotting

The radio immuno precipitation assay lysate was added to cardiomyocytes in each group to extract the protein. Protein concentration was then measured using a bicinchoninic acid assay protein concentration detection kit. An appropriate amount of protein loading buffer was then added, and electrophoresis separation was performed on SDS-PAGE gel. Proteins were then transferred onto PVDF membranes by transmembrane. After cutting according to the molecular size, the primary antibody was incubated. Following sealing, the membranes were incubated overnight at 4 °C with primary antibodies specific to the following antigens: β-Actin (dilution, 1:1000), ACSL4 (dilution, 1:1000), GPX4 (dilution, 1:1000), AMPK (dilution, 1:1000), p-AMPK (dilution, 1:1000), mTOR (dilution, 1:500), p-mTOR (dilution, 1:500). The membranes were then incubated with goat anti-rabbit IgG for 2 h (dilution, 1:20,000) to detect specific reaction products. Following the development of electrochemiluminescence solution, ImageJ Lab v4.0 was used to collect images and analyze the gray value of protein bands (*n* = 3).

### Statistical analysis

All the experiments were conducted in parallel three times. All data are expressed as the mean ± standard deviation of the results of three independent experiments. Differences between the two groups were compared using a paired t-test. One-way ANOVA was used to compare groups. *P* < 0.05 was considered to indicate a statistically significant difference. Data were analyzed using GraphPad Prism software 2022 (GraphPad Software, Inc.).

## Results

### Prediction of the biological processes, core targets and signaling pathways of imperatorin in the network pharmacology-based treatment of H/R

The TCMSP database was used to screen 22 bioactive components in *Angelica dahurica* (Table 1). Using Venny screening, 141 ferroptosis and *Angelica dahurica* intersection targets were obtained (Fig. 1B). Using the STRING database, a PPI network of 140 nodes and 660 interaction lines was obtained (Fig. 1C). Network visualization was also carried out using Cytoscape (Fig. 1D). These results indicated that the top three active components of *Angelica dahurica* were mandenol, alloisoimperatorin and ammidin. Only ACSL4 was found to be highly correlated with myocardial H/R injury in the intersection targets of *Angelica dahurica* and ferroptosis. In order to clarify the biological processes in which the targets of ferroptosis treatment by *Angelica dahurica* were involved, the present study analyzed the gene function of GO biological process of 141 potential targets using the DAVID database. These results suggested that *Angelica dahurica* treated ferroptosis through biological processes such as anti-apoptosis and anti-lipid metabolism (Fig. 1E-G). This study used the DAVID database for KEGG enrichment analysis of 141 key targets in order to explore the key signaling pathways in the intersection targets of *Angelica dahurica* and ferroptosis (Fig. 1H). The results showed that the insulin and AMPK signaling pathways were key signaling pathways, the former of which was not investigated in this study. The AMPK signaling pathway was therefore selected for subsequent test verification. By searching PDBID and its crystal structure used to molecular docking. The present study selected core targets (ACSL4, AMPK and mTOR) and core active ingredients (ammidin, octadecadiene and alloisoimperatorin). With AutoDock Vina molecular docking, the absolute value of the docking fraction represented conformational stability and affinities between the components and targets. The absolute value of > 4.25 some binding activity, that of > 5.0 indicated good binding activity and that of > 7.0 a strong binding activity. The results showed that AMPK and mTOR exhibited strong binding activities with ammidin (8.6 and 9.2, respectively), and that ACSL4 exhibited a strong binding activity with alloisoimperatorin (8.2). The binding energy of each active component was stronger than that of octadecadiene. ACSL4, AMPK and mTOR have a strong affinity with ammidin and alloisoimperatorin (Table 2). Comprehensive analysis showed that the docking binding energy of AMPK and ammidin was − 8.6 kcal/mol, that of ACSL4 and ammidin was − 7.7 kcal/mol and that of mTOR and ammidin was − 9.2 kcal/mol. Ammidin had strong binding activities with ACSL4, AMPK and mTOR. Specific binding patterns of target proteins and components were processed and optimized using PyMoL2.3.0 (Fig. 1I-K).

**Table 1:** A total of 22 bioactive ingredients of *Angelica dahurica* were screened using the TCMSP database (OB, ≥30%; DL, ≥0.18)

Herbal name	TCMSP ID	Compound	OB	DL
Baizhi	MOL001494	Mandenol	42	0.19
Baizhi	MOL001939	Alloisoimperatorin	34.8	0.22
Baizhi	MOL001941	Ammidin	34.55	0.22
Baizhi	MOL001942	isoimperatorin	45.46	0.23
Baizhi	MOL001956	Cnidilin	32.69	0.28
Baizhi	MOL002883	Ethyl oleate(NF)	32.4	0.19
Baizhi	MOL005789	Neobyakangelicol	36.18	0.31
Baizhi	MOL005792	(5-[2′(R)-Hydroxy-3′-methyl-3′-butenyl-oxy]furocoumarin)	42.85	0.26
Baizhi	MOL005800	Byakangelicol	41.42	0.36
Baizhi	MOL005802	Propyleneglycol monoleate	37.6	0.26
Baizhi	MOL005806	4-[(2S)-2,3-dihydroxy-3-methylbutoxy]furo[3.2-g]chromen-7-one	39.99	0.29
Baizhi	MOL005807	Sen-byakangelicol	58	0.61
Baizhi	MOL000358	beta-sitosterol	36.91	0.75
Baizhi	MOL000449	Stigmasterol	43.83	0.76
Baizhi	MOL000953	CLR	37.87	0.68
Baizhi	MOL001506	Supraene	33.55	0.42
Baizhi	MOL001749	ZINC03860434	43.59	0.35
Baizhi	MOL002644	Phellopterin	40.19	0.28
Baizhi	MOL003588	Prangenidin	36.31	0.22
Baizhi	MOL003791	Linolein,2-mono-	37.28	0.3
Baizhi	MOL007514	Methylicosa-11,14dienoate	39.67	0.23
Baizhi	MOL013430	Prangenin	43.6	0.29

**Table 2:** Binding abilities between drug components of *Angelica dahurica* and target proteins

Protein	PDBID	Ligand	Binding Affinity
AMPK	6C9H	Ammidin	−8.6
AMPK	6C9H	Mandenol	−5.7
AMPK	6C9H	Alloisoimperatorin	−8.5
mTOR	4DRI	Ammidin	−9.2
mTOR	4DRI	Mandenol	−6.7
mTOR	4DRI	Alloisoimperatorin	−8.9
ACSL4	AlphaFold2	Ammidin	−7.7
ACSL4	AlphaFold2	Mandenol	−5.7
ACSL4	AlphaFold2	Alloisoimperatorin	−8.2

Note: The absolute value of > 4.25 indicated certain binding activity, that of > 5.0 good binding activity and that of > 7.0 a strong binding activity

## Myocardial cell purity and effects of Na_2_S_2_O_4_ concentrations on cardiomyocytes

**Fig. 2:**
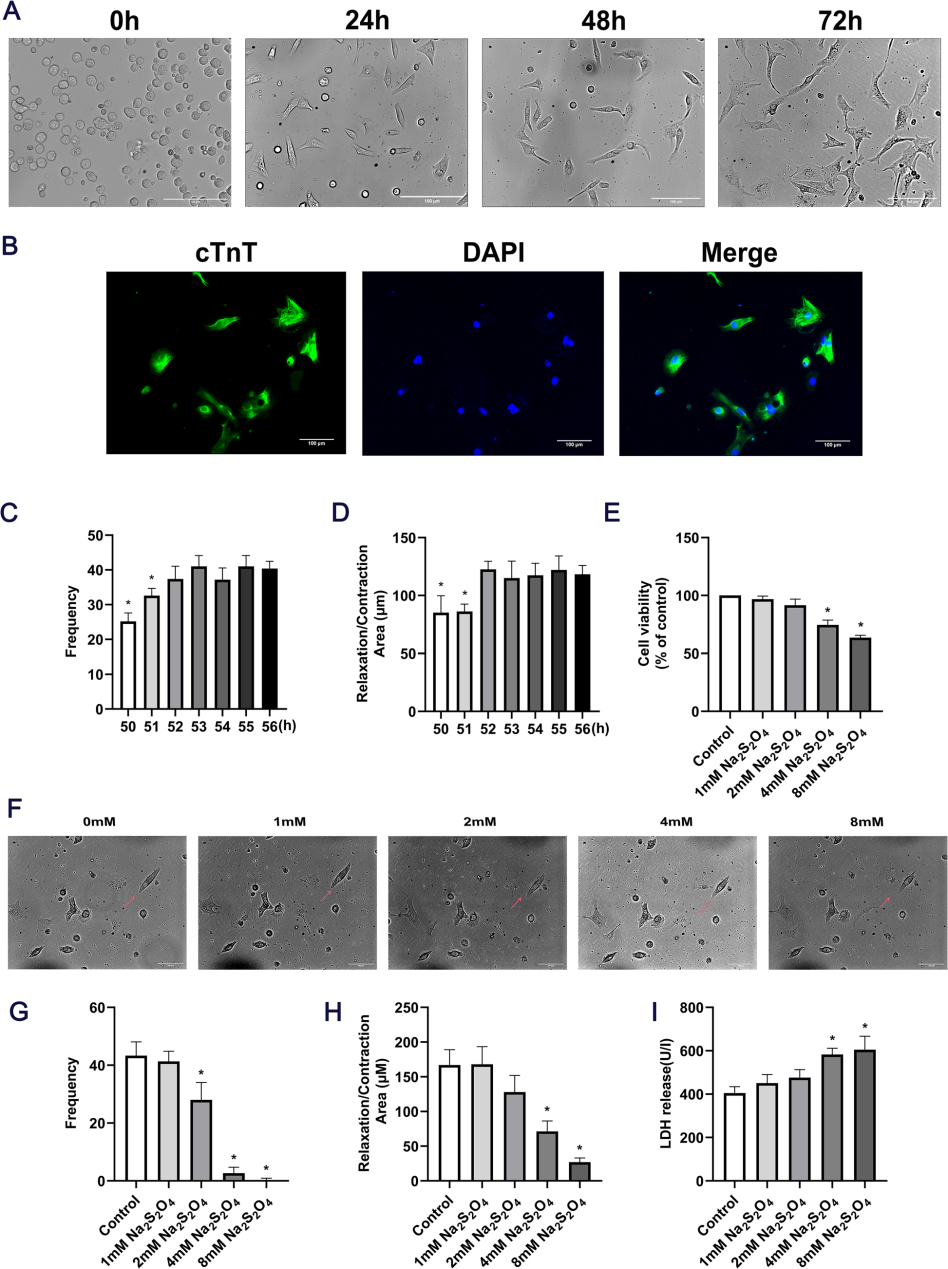
Determination of the beating frequency and cardiomyocyte area at different time points (50–56 h), and effects of Na_2_S_2_O_4_ concentrations (0, 1, 2, 4, 8 mM) on cardiomyocytes after a 1-h treatment. (**A**) Morphology under light microscopy at 0–72 h. (**B**) Immunofluorescence staining: Merge: a combination of cTnT-positive cells (green fluorescence) and nuclear DAPI staining (blue fluorescence). (**C**) Beating frequency during culture for 50–56 h. (**D**) Diastolic and contractile area during culture for 50–56 h. (**E**) Viability of cardiomyocytes. (**F**) Changes in cell morphology (cells that were clearly changing were marked by arrows). (**G**) Cell beating frequency. (**H**) Cell diastolic and contractile area. (**I**) LDH Release rate (^*^P < 0.05 vs. control; scale bars, 100 μm; *n* = 3). Na_2_S_2_O_4_, sodium dithionite; DAPI, 4′,6-diamidino-2-phenylindole; LDH, lactate dehydrogenase; cTnT, cardiac troponin T

The cells were round-shaped and began to adhere to the wall at 0–5 h, as shown under an inverted microscope. After 24 h, the cells had adhered to the wall, most of which showed spontaneous pulsation. After 48 h, the outstretched pseudopodia wound each other into a network. The pulsation tended to be synchronized. After 72 h, cell clusters were gradually formed and beat synchronously, resulting in functional syncytium (Fig. 2A). Cardiomyocytes were identified through a cTnT immunofluorescence assay with a purity of > 90% (Fig. 2B). The beating frequency (Fig. 2C) and area (Fig. 2D) of normal cardiomyocytes increased with time at 50–52 h (*P* < 0.05). The beating frequency and cardiomyocyte area tended to be stable at 52–56 h (*P* > 0.05). In conclusion, the primary cardiomyocytes were successfully extracted. The suitable experimental period was 52–56 h. As an oxygen-depleting agent, Na_2_S_2_O_4_ can rapidly consume oxygen in cell culture medium without damaging myocardial cell membranes, eventually creating an anoxic environment for cells. Following treatment with different concentrations of Na_2_S_2_O_4_ (0, 1, 2, 4 and 8 mM) for 1 h, the viability of primary cardiomyocytes was significantly decreased when treated with 4 mM Na_2_S_2_O_4_ (*P* < 0.05; Fig. 2E) as compared those that received control treatment, the cell morphology changed (Fig. 2F), the beating frequency of cardiomyocytes (Fig. 2G) and systolic area (Fig. 2H) were significantly decreased, and the release of LDH was significantly increased (Fig. 2I) (P < 0.05). These results showed that 4 mM Na_2_S_2_O_4_ had the most obvious effect following treatment of anoxia; thus 4 mM Na_2_S_2_O_4_ was selected for subsequent experiments.

### Effects of 4 mM Na_2_S_2_O_4_ on cardiomyocytes with different duration of hypoxia/reoxygenation durations

**Fig. 3:**
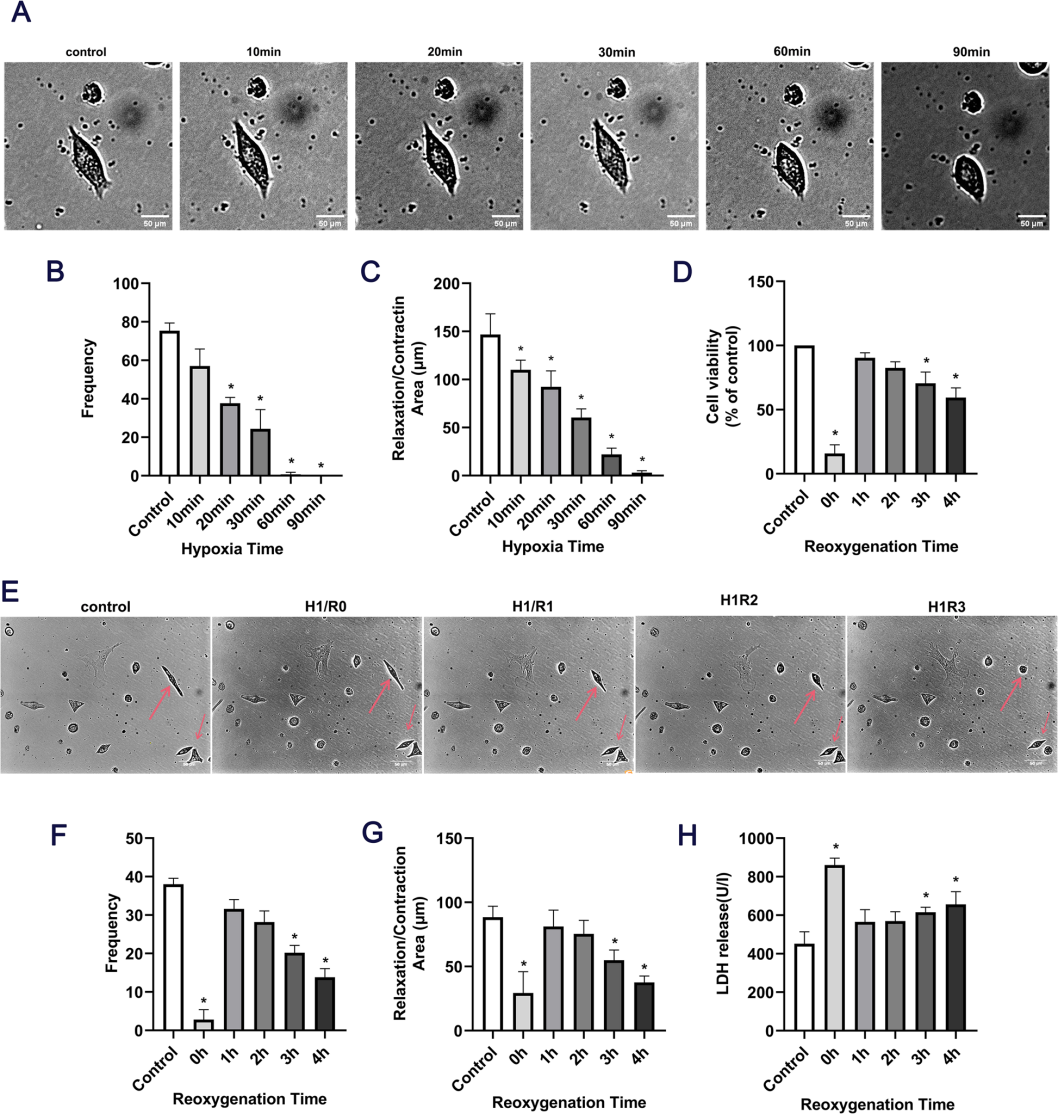
Effects of hypoxia for 10, 20, 30, 60 and 90 min / reoxygenation for 1, 2, 3 and 4 h on cardiomyocytes. (**A**) Changes in cell morphology. (**B**) Cell beating frequency. (**C)** Diastolic and contractile area. (**D**) Cardiomyocyte viability. (**E**) Changes in cell morphology (cells that were clearly changing were marked by arrows). (**F**) Cell beating frequency. (**G**) Diastolic and contractile area. (**H**) LDH release. (^*^P < 0.05 vs. control; ^#^P < 0.05 vs. H/R; *n* = 3). LDH, lactate dehydrogenase

Following the treatment of cardiomyocytes with 4 mM Na_2_S_2_O_4_ and hypoxia at different time points (10, 20, 30, 60 and 90 min), the beating frequency of the same cardiomyocyte was ~ 54 times/min after 10 min of hypoxia, as compared with the control group (beating frequency ~ 76 times/min). After 20 min of hypoxia, the beating frequency was ~ 37 times/min, after 30 min of hypoxia it was ~ 28 times/min, after 60 min of hypoxia, it was ~ 0 time/min, and after 90 min of hypoxia the cells ruptured and died (Fig. 3A-C). These results indicated that hypoxia had the most obvious effect on cells at 60 min (*P* < 0.05). Thus, cardiomyocytes were selected for 60 min of hypoxia for the follow-up experiments. Compared with the control group, cell viability decreased significantly under hypoxia for 1 h and reoxygenation for 3 h (Fig. 3D), and the cardiomyocyte morphology (Fig. 3E), beating frequency (Fig. 3F) and the diastolic and contractile area (Fig. 3G) were significantly decreased (*P* < 0.05). By contrast, the release of LDH was significantly increased (P < 0.05, Fig. 3H). These results indicated that the effect of hypoxia for 1 h and reoxygenation for 3 h was the most obvious (*P* < 0.05). Thus, 4 mM Na_2_S_2_O_4_ was used for hypoxia for 1 h and reoxygenation for 3 h in the follow-up experiments.

### Fer-1 effectively inhibits ferroptosis in cardiomyocytes

**Fig. 4:**
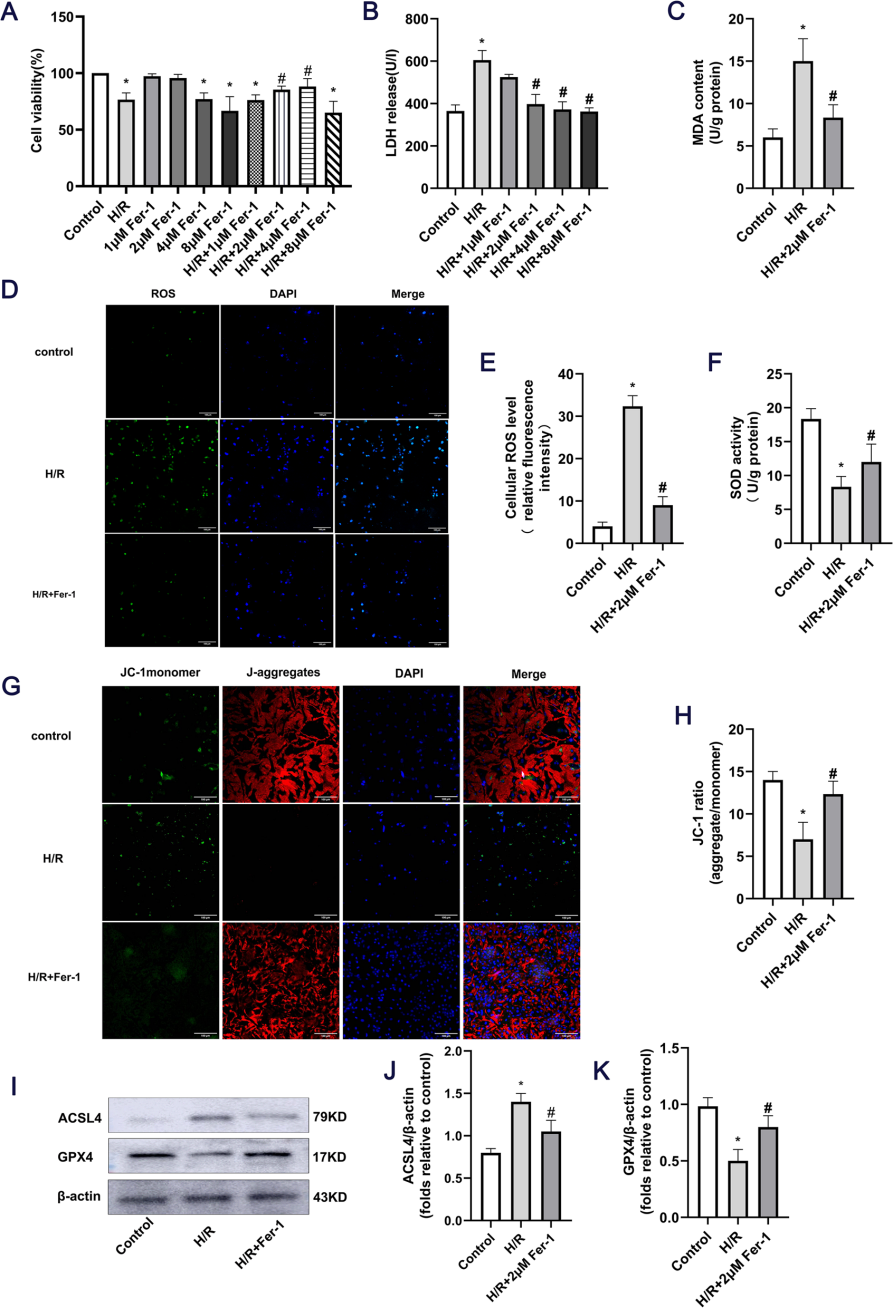
Effect of 2 μM Fer-1 on ROS levels in H/R-induced cardiomyocytes. (**A**) Cardiomyocyte viability. (**B**) LDH release. (**C**) MDA content. (**D**) ROS fluorescence image, Merge: A combination of ROS (green fluorescence) and nucleus (blue fluorescence). (**E**) ROS quantitative analysis. (**F**) SOD content. (**G**) JC-1 staining showed that JC-1 aggregated in the mitochondria of normal cells emitted red fluorescence, while JC-1 in dead cells emitted green fluorescence due to its presence in the cytoplasm as a monomer. Merge: A combination of aggregates (red fluorescence), monomers (green fluorescence) and nucleus (blue fluorescence). (**H**) Red/green fluorescence ratio at the MMP level. (**I-K**) Western blotting revealing the expression levels of ACSL4 and GPX4 in cardiomyocytes (^*^P < 0.05 vs. control; ^#^P < 0.05 vs. H/R; scale bars, 100 μm; *n* = 3). Proteins were then transferred onto PVDF membranes. After cutting according to the molecular size, the primary antibody was incubated. Fer-1, ferrostatin-1; ROS, reactive oxygen species; H/R, hypoxia/reoxygenation; LDH, lactate dehydrogenase; MDA, malondialdehyde; SOD, superoxide dismutase; JC-1, 1,1′,3,3′-tetraethyl-5,5′,6,6′-tetrachloroimidacarbocyanine iodide; MMP, mitochondrial membrane potential; ACSL4, Acyl-CoA synthetase long chain family member 4; GPX4, glutathione peroxidase 4

It has been reported that Fer-1 can specifically verify the existence of ferroptosis [16]; therefore, cardiomyocytes were treated with different concentrations of Fer-1 (1, 2, 4 and 8 μM) and no statistical significance in cell viability was observed between the control group and the control + 2 μM Fer-1 group (*P* < 0.05). However, in the control + 4 μM Fer-1 group, the cell viability was significantly decreased (*P* < 0.05), indicating that 2 μM Fer-1 was not toxic to normal cells. Compared with the model group, the cell viability in the H/R + 2 μM Fer-1 and H/R + 4 μM Fer-1 groups was significantly increased (*P* < 0.05). No statistical significance was observed in cell viability in the H/R + 8 μM Fer-1 group (P < 0.05, Fig. 4A). LDH release in the H/R + 2 μM Fer-1, H/R + 4 μM Fer-1 and H/R + 8 μM Fer-1 groups was significantly decreased (P < 0.05; Fig. 4B). The above results confirmed that the H/R + 2 μM Fer-1 group had statistically significant influence and did little damage to normal cells. Thus, 2 μM Fer-1 was selected for follow-up experiments. To further verify the occurrence of ferroptosis in myocardial H/R injury, variations in other ferroptosis-related indicators were also examined. The results showed that, compared with the model group, MDA release in the H/R + 2 μM Fer-1 group was decreased (P < 0.05; Fig. 4C), ROS levels were significantly decreased (*P* < 0.05; Fig. 4D-E), SOD release was increased (*P* < 0.05; Fig. 4F), the red/green fluorescence ratio of mitochondrial JC-1 returned to normal (P < 0.05; Fig. 4G-H), GPX4 protein expression was decreased (P < 0.05) and ACSL4 protein expression was increased (P < 0.05; Fig. 4I-K). These results confirmed the occurrence of ferroptosis in myocardial H/R injury.

### Effects of ammidin and ACSL4 inhibitor (PRGL493) concentrations on cardiomyocytes

**Fig. 5:**
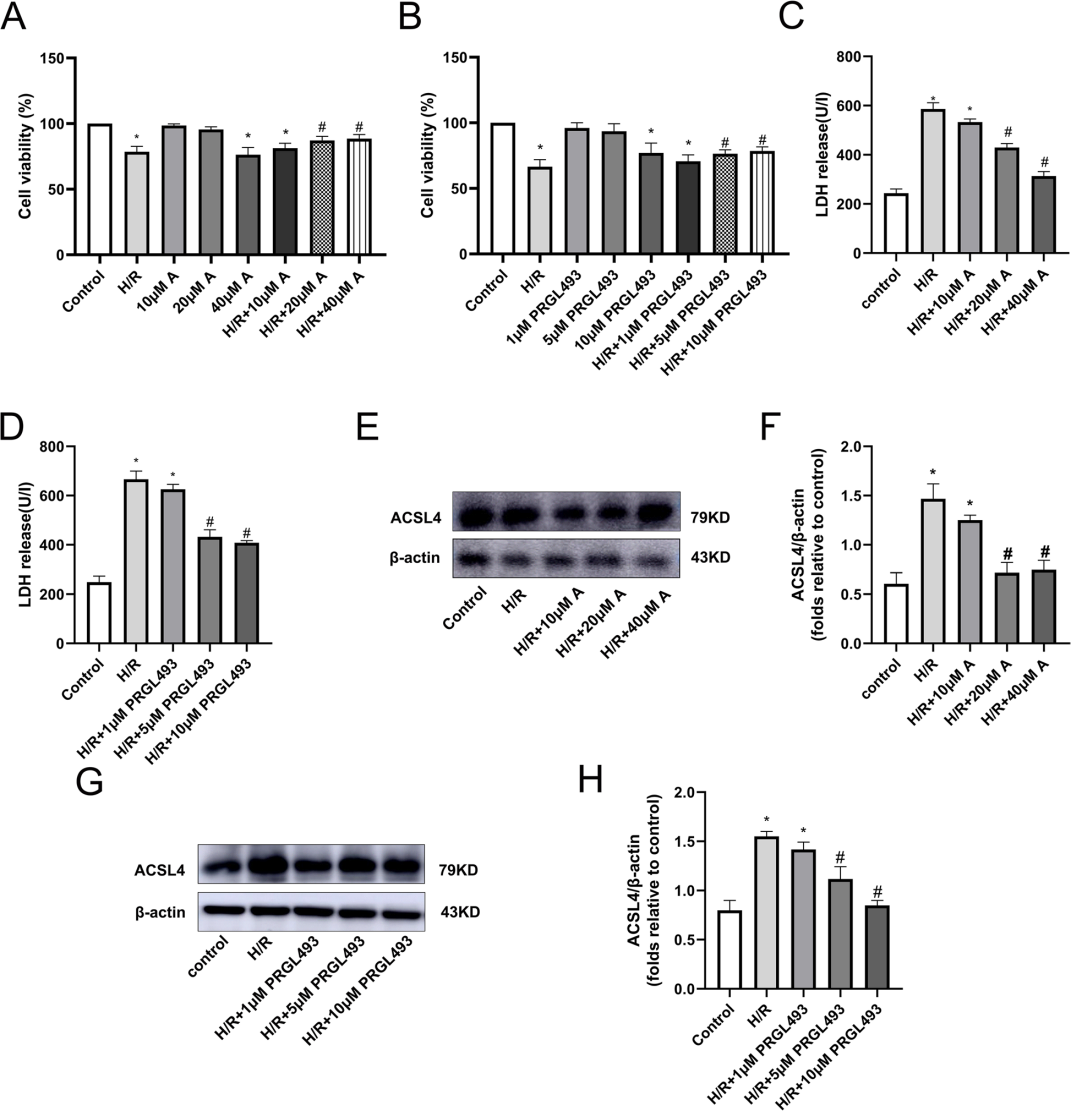
Effects of ammidin (0, 10, 20 and 40 μM) and PRGL493 concentrations (0, 1, 5 and 10 μM) on cardiomyocytes in the normal and H/R groups. (**A-B**) Cardiomyocyte viability. (**C-D**) LDH release. (E-H) ACSL4 protein expression (^*^P < 0.05 vs. control; ^#^P < 0.05 vs. H/R; *n* = 3). Proteins were then transferred onto PVDF membranes. After cutting according to the molecular size, the primary antibody was incubated. H/R, hypoxia/reoxygenation; LDH, lactate dehydrogenase; ACSL4, Acyl-CoA synthetase long chain family member 4

Cardiomyocytes from each group were pretreated with ammidin (0, 10, 20 and 40 μM) and PRGL493 (0, 1, 5 and 10 μM). The Fig. 5 results showed that, compared with the control group, there was no statistical significance in the cell viability of the 10 μM ammidin, 20 μM ammidin, 1 μM PRGL4935 and 5 μM PRGL493 groups (*P* < 0.05), while the cell viability of the 40 μM ammidin and 10 μM PRGL493 groups was significantly decreased (P < 0.05). The results suggested that 10 μM ammidin, 20 μM ammidin, 1 μM PRGL493 and 5 μM PRGL493 had no toxicity to normal cells. Meanwhile, as compared with the model group, cell viability in the H/R + 20 μM ammidin, H/R + 40 μM ammidin, H/R + 5 μM PRGL493 and H/R + 10 μM PRGL493 groups was increased (*P* < 0.05; Fig. 5A-B), LDH release was decreased (P < 0.05; Fig. 5C-D) and ACSL4 protein expression was also decreased (*P* < 0.05; Fig. 5E-H). The results showed that the H/R + 20 μM ammidin and H/R + 5 μM PRGL493 groups had a statistically significant influence and did little damage to normal cells, and were therefore selected for subsequent experiments.

### Ammidin alleviated ferroptosis-induced myocardial H/R injury by reducing the expression of ACSL4 to regulate the AMPK/mTOR pathway

**Fig. 6:**
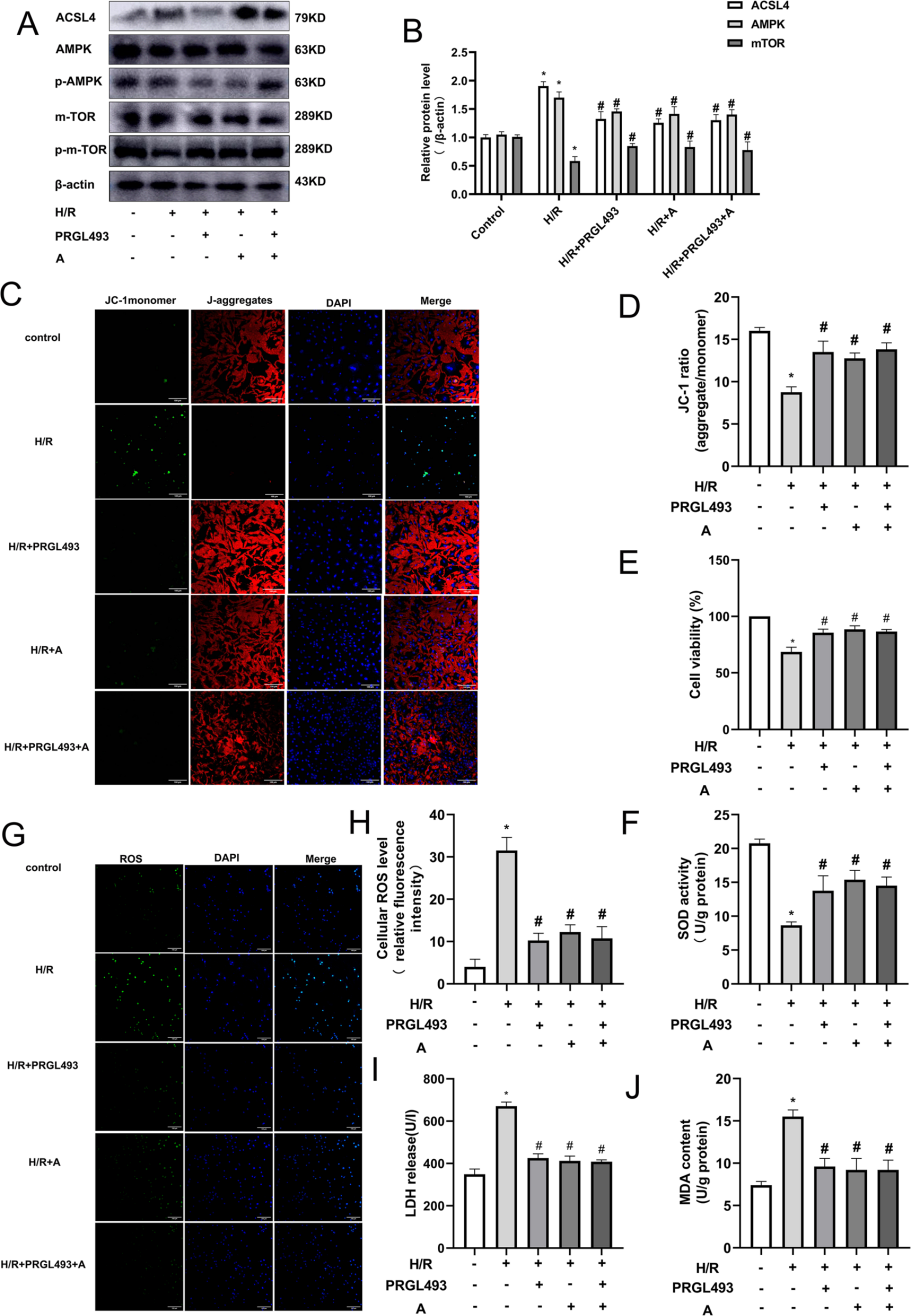
Variations in cardiomyocytes in each group treated with 5 μM PRGL493 and 20 μM ammidin. (**A-B**) Protein expression of ACSL4, AMPK, p-AMPK, mTOR and p-mTOR. Proteins were then transferred onto PVDF membranes. After cutting according to the molecular size, the primary antibody was incubated. (**C-D**) JC-1 staining showed that JC-1 aggregated in the mitochondria of normal cells emitted red fluorescence, while JC-1 in dead cells emitted green fluorescence due to its presence in the cytoplasm as a monomer. Merge: A combination of aggregate (red fluorescence), monomer (green fluorescence) and nucleus (blue fluorescence). (**E**) Cardiomyocyte viability. (**F**) SOD level. (G-H) ROS fluorescence image, Merge: A combination of ROS (green fluorescence) and nucleus (blue fluorescence). (**I**) LDH release rate. (**J**) MDA level. (^*^P < 0.05 vs. control. ^#^P < 0.05 vs. H/R; scale bars: 100 μm; *n* = 3). ACSL4, Acyl-CoA synthetase long-chain family member 4; p-, phosphorylated; SOD, superoxide dismutase; ROS, reactive oxygen species; LDH, lactate dehydrogenase; MDA, malondialdehyde; H/R, hypoxia/reoxygenation;JC-1,1,1′,3,3′-tetraethyl-5,5′,6,6′-tetrachloroimidacarbocyanine iodide

Western blotting was performed to explore whether ammidin has a regulatory effect on the AMPK/mTOR pathway. The results showed that, as compared with the control group (Fig. 6A-B), the expression levels of the ACSL4 and AMPK protein in the H/R group increased, while that of mTOR protein decreased (*P* < 0.05). The expression levels of ACSL4 and AMPK in the ACSL4 inhibitor PRGL493 group were decreased, while those of mTOR protein were increased (P < 0.05). The expression levels of ACSL4 and AMPK in the ammidin group were decreased, while those of mTOR protein were increased (P < 0.05); the expression levels of ACSL4 and AMPK in the PRGL493 + ammidin group were decreased, while those of mTOR protein were increased (*P* < 0.05). The results confirmed that ammidin could regulate the AMPK/mTOR pathway by reducing the expression of ACSL4. As compared with the model group, mitochondrial JC-1 red/green fluorescence ratio, cell activity and SOD levels were significantly increased in the H/R + PRGL493, H/R + ammidin and H/R + PRGL493 + ammidin (P < 0.05; Fig. 6C-F) groups, and the levels of ROS, LDH and MDA were significantly decreased (P < 0.05; Fig. 6G-J). The results confirmed that ammidin could inhibit the activation of the AMPK/mTOR pathway by reducing the expression of ACSL4, thus alleviating the ferroptosis-induced myocardial H/R injury.

## Discussion

In recent years, the incidence and mortality rates of myocardial H/R injury have gradually increased [17, 18]. It was found that *Angelica dahurica* has pharmacological activities, such as anti-apoptosis, anti-inflammation and anti-oxidation activities [19]. Network pharmacology was used based on OB and DL, and predicted that the main chemical components of *Angelica dahurica* were mannitol, isoprofloxacin and aminobutyl, among which aminobutyl was the main component with a cardiovascular protection effect. However, no reports have been found on the association between ammidin and ferroptosis. Therefore, the interaction network between ammidin and the intersection target of ferroptosis was analyzed using the STRING database [20]. It was found that ACSL4 was the player in ammidin-induced ferroptosis inhibition, and the biological functions and signaling pathways of intersection targets were analyzed by GO and KEGG pathway enrichment analysis, indicating that the AMPK signaling pathway was the key signaling pathway. Finally, Autodock software was used to verify the molecular docking of core components and targets. The results showed that ammidin had a strong binding activity with ACSL4, AMPK and mTOR. Based on the above prediction of the biological processes, core target and core pathway of ammidin against ferroptosis, we speculated that ammidin has a therapeutic effect on cardiomyocyte H/R, which may be enacted through the inhibition of ferroptosis and regulation of the ACSL4/AMPK signaling pathway.

Na_2_S_2_O_4_ is a strong reducing agent, which can be rapidly consumed without damaging the myocardial cell membrane, resulting in an anoxic environment for cardiomyocytes. In the experiment, a myocardial cell H/R injury model established by Na_2_S_2_O_4_ was used [21]. The decrease in cell survival rate, as well as the change in beating frequency and systolic and diastolic amplitude of cardiomyocytes were the typical manifestations of H/R injury in cardiomyocytes. LDH release was positively correlated with the degree of myocardial cell injury [22]. In the present study, 4 mM Na_2_S_2_O_4_ was used to simulate myocardial cell H/R injury model. The survival rate of cardiomyocytes was determined by CCK-8 method, LDH release was determined by ultraviolet spectrophotometry, and the pulsation frequency and systolic and diastolic amplitude changes in cardiomyocytes were recorded by living cell workstation. The results showed that, compared with the control group, the activity of cardiomyocytes in the H/R group was decreased, the LDH level was increased, and the contractile amplitude and diastolic difference of cardiomyocytes were decreased. The results showed that the myocardial cell H/R injury model was successfully replicated in this experiment.

In 2012, Doxin et al [23] formally proposed ferroptosis. Ferroptosis is characterized by intracellular iron accumulation, lipid peroxidation and ROS accumulation [24]. ROS is a type of oxygen-containing active substances with a high reactivity [25]. Excessive ROS can lead to lipid peroxidation in the cell membrane and antioxidant system disorder [26]. SOD catalyzes the disproportionation of superoxide anion radicals into hydrogen peroxide and oxygen, which is an important oxygen radical scavenger [27]. MDA can reflect the degree of oxidative damage. Prolonged ischemia and hypoxia reduced MMP and activated the mitochondrial apoptotic pathway, leading to the further destruction of ischemic cells. Therefore, MMP, ROS, MDA and SOD can be used as important indicators of ferroptosis. In the present study, primary cardiomyocytes were used to establish an H/R injury model, and the changes in the above indices were observed. The results showed that during H/R injury, SOD release was increased, MDA release and ROS production was decreased, and the mitochondrial membrane permeability conversion hole opened, leading to a decrease in MMP. Fer-1 is an antioxidant that can effectively inhibit ferroptosis-induced cell damage [28]. The present study found that, compared with the H/R group, ACSL4 protein expression, a key indicator of ferroptosis, was decreased following the addition of Fer-1, while GPX4 protein expression, myocardial cell survival rate, SOD levels and MMP levels were increased, and LDH, MDA and ROS levels were decreased, indicating that Fer-1 could reduce lipid peroxide deposition. Thus, Fer-1 can inhibit the generation of oxidative stress and further protect cardiomyocytes. These results indicated that ferroptosis occurs during the H/R generation of cardiomyocytes.

According to Song et al [29], the key regulatory proteins of ferroptosis include ACSL4 and GPX4. The western blotting results showed that ACSL4 expression was increased and GPX4 was decreased during the H/R injury process, suggesting that ferroptosis was caused by changes in ACSL4 and GPX4 contributed to the occurrence and development of H/R injury in cardiomyocytes.

ACSL4 overexpression induces the activation of the ferroptosis pathway. Xu et al [30] showed that ACSL4 silencing reduced lipid peroxidation and increased GSH and GPX4 expression, thus inhibiting ferroptosis and reducing lung H/R injury. A previous study established a model of myocardial hypoxia and reoxygenation injury, which proved that ACSL4 overexpression could induce ferroptosis and aggravate myocardial injury [31]. As a central energy metabolism switch, AMPK plays a crucial role in cell functional processes, such as cell proliferation, death and survival [32]. AMPK inhibits acetyl-coA carboxylase-mediated polyunsaturated fatty acid biosynthesis and maintains mitochondrial homeostasis to inhibit ferroptosis [33]. Cheng et al [12] demonstrated that ACSL4 promoted sevofluran-induced ferroptosis in neurons by inhibiting the AMPK/mTOR signaling pathway, and ACSL4 inhibition or knockdown could play a protective role in sevofluran-induced ferroptosis by activating the AMPK/mTOR signaling pathway. These experimental results showed that the administration of ACSL4 inhibitor and ammidin treatment reduced the occurrence of ferroptosis during myocardial cell H/R injury; among its indicators, cell viability, and SOD and MMP contents were increased, and ROS levels, and LDH and MDA contents were decreased. These results were consistent with those of previous studies, suggesting that ACSL4 silencing alleviates myocardial injury by inhibiting the occurrence of ferroptosis. Meanwhile, in the H/R injury group, the expression of ACSL4 and AMPK protein increased, while that of mTOR protein decreased, indicating that the activation of the ACSL4/AMPK/mTOR signaling pathway was inhibited. This inhibition was reduced in the ACSL4 inhibitor group, which was the same following ammidin treatment. These results indicated that ammidin could inhibit ferroptosis by regulating the ACSL4/AMPK/mTOR signaling pathway and improve myocardial cell H/R injury, which was consistent with the predicted results of network pharmacology.

In conclusion, this study successfully replicated the H/R injury model of cardiomyocytes. Fer-1, an ferroptosis inhibitor, inhibits the production of intracellular ROS by regulating ACSL4 and GPX4, thus significantly increasing the survival rate and MMP level of cardiomyocytes and subsequently alleviating the ferroptosis-induced H/R injury of primary cardiomyocytes. This indicates that ferroptosis occurs during the H/R injury of cardiomyocytes. It was predicted through network pharmacology that ammidin has a therapeutic effect on cardiomyocyte H/R, and the mechanism may be the inhibition of ferroptosis and regulation of the ACSL4-AMPK signaling pathway. In vitro experiments also demonstrated that ammidin treatment plays a protective role in H/R injury in cardiomyocytes, which may be mediated by the inhibition of ferroptosis during H/R injury by regulating the ACSL4/AMPK/mTOR signaling pathway. The results of the present study can provide a new drug target for the treatment of myocardial cell H/R injury.

## Conclusion

In conclusion, primary myocardial cells from lactating mice were used in the present study. Based on network pharmacology, the correlation between ammidin and ferroptosis was predicted. In vitro experiments were conducted to verify the occurrence of ferroptosis in cardiomyocyte H/R injury, and it was confirmed that ammidin may regulate the ACSL4/AMPK/mTOR signaling pathway to reduce the ferroptosis-induced cardiomyocyte H/R injury and play a therapeutic role in cardiomyocyte H/R injury. However, the present study had certain limitations, and we plan to further verify this conclusion through animal experiments.
